# Supplementary material for: Central insulin modulates food valuation via mesolimbic pathways
Source: Nat Commun. 2017 Jul 18;8:16052. doi: 10.1038/ncomms16052 (PMC5520049; doi:10.1038/ncomms16052)
Supplement: Supplementary Information [file ncomms16052-s1.pdf]

Title of file for HTML: Supplementary Information
Description: Supplementary Figure, Supplementary Tables, Supplementary Methods and Supplementary References

Title of file for HTML: Peer Review File
Description:

SUPPLEMENTARY FIGURES:

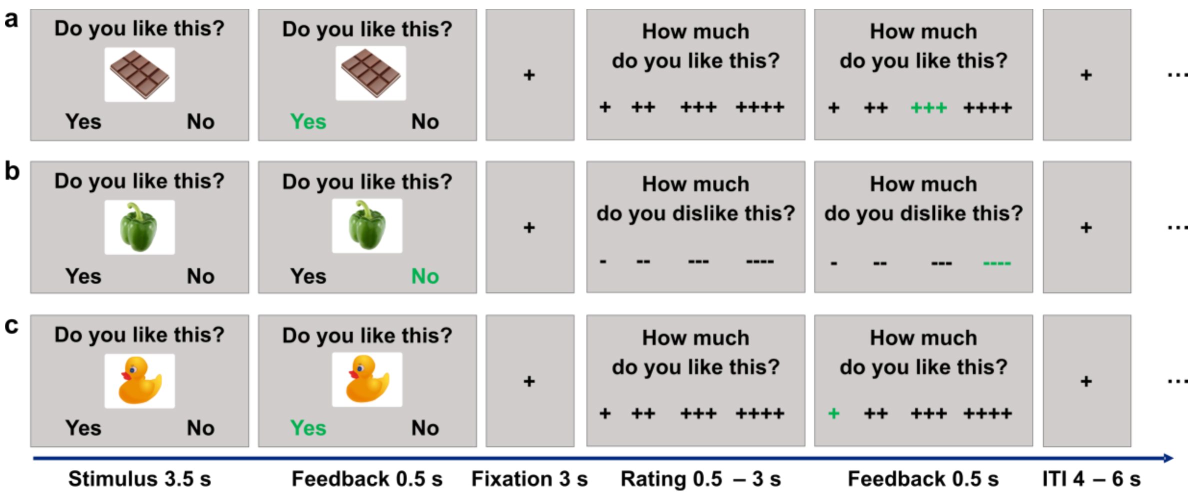

Supplementary Figure 1 | Paradigm. Each of the two sets (see Methods and Supplementary Table 1) consisted of 70 food stimuli (**a**, **b**), as well as 70 non-food stimuli (**c**). Amongst the food stimuli, there were both palatable **(a)** as well as rather unpalatable food items **(b)**. The participants were given feedback for their button presses by highlighting the selected answer in green. Following "yes" (**a**, **c**), participants were asked how much they like the item, following "no" (**b**), they were asked how much they dislike the item. In both cases, the scale was ordered by intensity, with four pluses representing the strongest like, and four minuses the strongest dislike.

SUPPLEMENTARY TABLES:

	Set 1	Set 2	*P*
**All items**			
Picture saliency	0.18 (0.001)	0.20 (0.001)	n.s.
Parametric liking score (validation study)	2.67 (0.05)	2.70 (0.04)	n.s.
**Food items**			
Picture saliency	0.19 (0.001)	0.20 (0.001)	n.s.
Parametric liking score (validation study)	2.86 (0.05)	2.94 (0.04)	n.s.
**Non-food items**			
Picture saliency	0.17 (0.001)	0.19 (0.001)	n.s.
Parametric liking score (validation study)	2.48 (0.04)	2.47 (0.04)	n.s.

Supplementary Table 1 | Stimuli characteristics. In a validation study, an independent sample of 16 participants rated the preference of items on a scale from 1 (~ "I do not like this at all") to 4 (~ "I like this very much").

Saliency is calculated based on the Image Signature algorithm, as described by Hou et al. [1].

This approach calculates the saliency map of an image by the identification of visually conspicuous image locations based on a discrete cosine transform (DCT) that transforms spatial to frequency signals.

The two sets did not differ in regard to the parametric liking score or the picture salience.

Values indicate means with s.e.m. in parentheses.

	Food categories (% liked/total)							
	Sweets	Salty snacks	Dairy products	Fast-food	Fruits	Baked goods	Tapas	Vegetables
**NIR**	.92 (.05)	.82 (.07)	.96 (.04)	.82 (.07)	.89 (.06)	.89 (.06)	.50 (.09)	.92 (.05)
**IR**	.90 (.07)	.85 (.08)	.85 (.08)	.90 (.07)	.95 (.05)	.85 (.08)	.65 (.11)	.90 (.07)
P	n.s.	n.s.	n.s.	n.s.	n.s.	n.s.	n.s.	n.s.

Supplementary Table 2 | General food preferences. To assess the general preference of different kind of foods, all food items were grouped into eight categories. Chi-Square tests revealed no significant differences in general food liking between the two groups. Consistently, there was no significant group x category interaction ($F_{(7,40)}$ = 1.468; P = .206, n_{NIR} = 28, n_{IR} = 20).

Values indicate group means of liked/all items within each food category with s.e.m. in parentheses. NIR: participants with normal insulin sensitivity, IR: insulin-resistant participants

a

	Fasting duration (hours)				
	**PL**	**IN**	***P***	***P*** (NIR vs. IR)	***P*** (group x session)
**NIR**	12.78 (.31)	12.75 (.28)	n.s.	n.s.	n.s.
**IR**	12.72 (.24)	13.08 (.29)	n.s.		

b

	Prior food intake (kcal)				
	**PL**	**IN**	***P***	***P*** (NIR vs. IR)	***P*** (group x session)
**NIR**	329.10 (31.28)	302.42 (27.42)	n.s.	n.s.	n.s.
**IR**	274.92 (22.66)	262.09 (33.66)	n.s.		

Supplementary Table 3 | (a) Fasting duration before each study day and (b) last caloric intake. Neither did the fasting times (a) between the last food intake and the beginning of the study day differ between groups or sessions, nor was there a group x session effect (all $P > .24$, $n_{NIR} = 28$, $n_{IR} = 20$, rmANOVA). The total caloric intake (b) of the protocolled last food intake before fasting was computed using the software DGExpert 1.8.6 (German Nutrition Society) for each participant on both scan days. Results revealed no significant group, session or group x session effect (all $P > .46$, $n_{NIR} = 28$, $n_{IR} = 20$, rmANOVA).

Values indicate means with s.e.m. in parentheses. NIR: participants with normal insulin sensitivity, IR: insulin-resistant participants, PL: placebo, IN: insulin

NIR	PL		*P*	IN		*P*	*P*
	**Pre**	**Post**		**Pre**	**Post**		**(interaction)**
Insulin (pmol/L)	40.14 (3.2)	35.42 (2.6)	< .05	41.25 (2.6)	45.56 (3.2)	n.s.	<.05
Glucose (mmol/L)	4.64 (0.1)	4.78 (0.1)	< .05	4.68 (0.1)	4.65 (0.1)	n.s.	n.s.

IR	PL		*P*	IN		*P*	*P*
	**Pre**	**Post**		**Pre**	**Post**		**(interaction)**
Insulin (pmol/L)	76.33 (7.1)	68.55 (7.0)	n.s.	77.02 (7.8)	66.50 (5.7)	< .05	n.s.
Glucose (mmol/L)	4.96 (0.1)	5.00 (0.1)	n.s.	4.86 (0.1)	4.86 (0.1)	n.s.	n.s.

Supplementary Table 4 | Pre-post blood values. Blood samples were collected after arrival and after completion of the scanning sessions (see Fig. 1a). Only in NIR individuals, there was a significant insulin level x session interaction driven by a stronger insulin decrease in the placebo session ($F_{(1, 27)}$ = 15.45; P = .001, η^2 = .364, n = 28, rmANOVA).

Values indicate means with s.e.m. in parentheses. NIR: participants with normal insulin sensitivity, IR: insulin-resistant participants, PL: placebo, IN: insulin

Brain region	Side	MNI (peak) *x*	*y*	*z*	Cluster Size	*Z*
**<u>Paradigm-induced activation patterns at placebo</u>**						
**Food > non-food**						
Middle frontal gyrus	L	-24	34	-13	636	Inf
	R	21	29	-19	693	Inf
Inferior frontal gyrus	L	-36	34	14	544	6.18
Superior frontal gyrus	R	4	34	40	846	5.85
Medial frontal gyrus	R	12	11	-18	Same cluster	5.19
Anterior insula	L	-38	5	-12	2523	Inf
	R	39	8	-12	2849	Inf
Amygdala	R	20	-1	-22	2849	7.07
Ventral tegmental area	L	-4	-13	-12	47	4.72
	R	6	-12	-13	15	3.93
Postcentral gyrus	L	-60	-19	32	1151	7.28
Middle cingulate gyrus	R	4	-19	30	1362	6.58
Posterior cingulate gyrus		0	-30	32	Same cluster	6.68
Inferior temporal gyrus	L	-56	-52	-19	544	7.21
Lingual gyrus	L	-12	-94	-4	757	5.45
**Parametric modulation by preference values**						
Superior frontal gyrus	L	-4	59	6	9527	Inf
Anterior cingulate gyrus	L	-10	47	-6	Same cluster	Inf
Nucleus accumbens	L	-10	8	-6	6	3.38
	R	9	8	-6	19	3.93
Middle temporal gyrus	L	-64	-49	-8	2294	Inf
Precuneus	L	-9	-60	16	1531	6.74
Cerebellum	R	45	-60	-40	576	5.97
Lingual gyrus	L	-12	-74	-12	813	5.15
**Parametric modulation, $NIR_{food>non\text{-}food}$ >$IR_{food>non\text{-}food}$**						
Nucleus accumbens	L	-12	8	-8	26	3.85
**<u>Neural insulin effects (PL > IN)</u>**						
**Parametric modulation, $NIR_{food>non\text{-}food}$ >$IR_{food>non\text{-}food}$**						
Nucleus accumbens	L	-12	8	-8	17	3.5
	R	10	8	-7	10	3.1
Ventral tegmental area	L	-4	-12	-14	7	3.13

Supplementary Table 5 | Peak coordinates and statistics of fMRI analyses. Montreal Neurological Institute (MNI) coordinates and z values are reported for peak voxels and local maxima within each cluster. All *P* < .05 FWE corrected, L: left, R: right, NIR: participants with normal insulin sensitivity, IR: insulin-resistant participants, PL: placebo, IN: insulin

	VTA → NAc	NAc → VTA	VTA intrinsic	NAc intrinsic
**L**				
**A (Hz)**	**0.05 (.02)**	**0.33 (.01)**	**-0.74 (.02)**	**-0.23 (.02)**
**B (Hz)**	**-0.49 (.09)**	0 (0)	**-1.73 (.12)**	**-1.97 (.10)**
**R**				
**A (Hz)**	-0.03 (.01)	0.02 (.02)	**-0.08 (.02)**	**-0.10 (.02)**
**B (Hz)**	**-0.23 (.05)**	0 (.06)	**-1.65 (.11)**	**-1.68 (.07)**

Supplementary Table 6 | DCM parameter estimates of the winning models in the left and right hemisphere. Mean parameter values of fixed-connections (A) and insulin modulation (B) in the winning model across all participants for the left and the right hemisphere. Bayesian Parameter Averages are shown with s.e.m. in parentheses. Values which are significantly different from zero (*P* < .007 Bonferroni-corrected for multiple comparisons) are printed in bold.

VTA: ventral tegmental area, NAc: nucleus accumbens, L: left, R: right, Hz: Hertz

SUPPLEMENTARY METHODS:

Peripheral and anthropometric measures

During fMRI scanning, measures of cardiac signals, recorded with a finger clip placed on the index finger of the left hand, and respiratory signals, recorded with a pressure belt placed around the umbilical region, were assessed to exclude session effects on peripheral body functions. Repeated measures ANOVAs yielded no significant effects of group or condition on heart rate or respiration (all $P > .10$; $n_{NIR} = 28$, $n_{IR} = 20$) (Fig. 3c, d).

Waist and hip circumference, height and weight anthropometric measures were taken after participants' arrival with normed scales according to a standard protocol. To measure hip circumference, a measuring tape was placed at the top of the hip bone (iliac crest), while the participant was standing upright. Waist circumference was assessed at the narrowest part of the waist. The body mass index (BMI) was calculated as the weight (kg) divided by the square of the height (meters). Body fat was assessed with a hand-hold electronic device measuring current flow through the upper body. Table 1 provides an overview of sample characteristics in NIR and IR groups.

Blood samples and analysis

Before application of intranasal insulin or placebo, 2.7 ml blood in a sodium fluoride tube for analysis of blood glucose, 7.5 ml blood in a serum tube for analysis of insulin, c-peptide, cortisol and leptin were collected. After completion of the MR scans, the collection of blood samples for insulin and glucose analysis was repeated. Blood samples were centrifuged at room temperature at 2,800g for ten minutes, the supernatants were stored at -80° C until further processing. Blood glucose was determined through photometry (Beckman Coulter);

plasma insulin, c-peptide and cortisol were measured with an electro-chemiluminescence immunoassay (Roche, ECLIA).

SUPPLEMENTARY REFERENCES:

1. Hou, X. *et al*. Image Signature: Highlighting sparse salient regions. *IEEE Trans. Pattern Anal. Mach. Intell*. **34,** 194-201 (2012).
